# Genotype-phenotype relationship in patients with arrhythmogenic right ventricular cardiomyopathy caused by desmosomal gene mutations: A systematic review and meta-analysis

**DOI:** 10.1038/srep41387

**Published:** 2017-01-25

**Authors:** Zhenyan Xu, Wengen Zhu, Cen Wang, Lin Huang, Qiongqiong Zhou, Jinzhu Hu, Xiaoshu Cheng, Kui Hong

**Affiliations:** 1 Department of Cardiovascular Medicine, the Second Affiliated Hospital of Nanchang University, Nanchang of Jiangxi, 330006, China; 2 Jiangxi Key Laboratory of Molecular Medicine, Nanchang of Jiangxi, 330006, China

## Abstract

The relationship between clinical phenotypes and desmosomal gene mutations in patients with arrhythmogenic right ventricular cardiomyopathy (ARVC) is poorly characterized. Therefore, we performed a meta-analysis to explore the genotype-phenotype relationship in patients with ARVC. Any studies reporting this genotype-phenotype relationship were included. In total, 11 studies involving 1,113 patients were included. The presence of desmosomal gene mutations was associated with a younger onset age of ARVC (32.7 ± 15.2 *versus* 43.2 ± 13.3 years; *P* = 0.001), a higher incidence of T wave inversion in V_1–3_ leads (78.5% *versus* 51.6%; *P* = 0.0002) or a family history of ARVC (39.5% *versus* 27.1%; *P* = 0.03). There was no difference in the proportion of males between desmosomal-positive and desmosomal-negative patients (68.3% versus 68.9%; *P* = 0.60). The presence of desmosomal gene mutations was not associated with global or regional structural and functional alterations (63.5% *versus* 60.5%; *P* = 0.37), epsilon wave (29.4% *versus* 26.2%; *P* = 0.51) or ventricular tachycardia of left bundle-branch morphology (62.6% *versus* 57.2%; *P* = 0.30). Overall, patients with desmosomal gene mutations are characterized by an earlier onset age, a higher incidence of T wave inversion in V_1–3_ leads and a strong family history of ARVC.

Arrhythmogenic right ventricular cardiomyopathy (ARVC) is an uncommon inherited myocardial disease with incomplete penetrance^1^, characterized by ventricular arrhythmias, progressive fibrofatty replacement and even sudden cardiac death (SCD)^2^. To date, 13 genes have been reported in patients with ARVC, and desmosomal genes account for more than 50% of all cases, including plakoglobin (JUP), plakophilin-2 (PKP2), desmoplakin (DSP), desmoglein-2 (DSG2), and desmocollin-2 (DSC2)^3^. Meanwhile, non-desmosomal genes are also implicated in ARVC, such as transmembrane protein 43, desmin, titin, phospholamban, and ryanodine receptor 2^4^,^5^,^6^. Recently, studies in patients with ARVC have shed light on the molecular mechanisms involved, and considerable progress has been made in the diagnosis and management of ARVC patients, but numerous challenges still exist due to the genetic and clinical heterogeneity in these patients^7^. Desmosomal gene mutations can lead to dysfunction of intercellular gap junction and electrical activity, conditions that are more vulnerable to mechanical stress^8^. As the most common variants, desmosomal gene mutations are closely related to several clinical characteristics, including T wave inversion, epsilon wave, onset age of ARVC and ventricular arrhythmias^9^,^10^,^11^. However, the definite genotype-phenotype relationship in patients with ARVC caused by desmosomal gene mutations is still poorly characterized. Moreover, data from previous studies on the clinical phenotypes of desmosomal-positive and desmosomal-negative patients are contradictory and limited due to the small population size. Therefore, we performed a meta-analysis of current studies on selected clinical features to explore the genotype-phenotype relationship in patients with ARVC caused by desmosomal gene mutations.

## Methods

The protocol for the analysis and reporting of the results of this systematic review and meta-analysis was based on the PRISMA (Preferred Reporting Items for Systematic reviews and Meta-Analyses) statement^12^.

### Literature search and study selection

We systematically searched the Cochrane Library, PubMed, and Elsevier databases through June 2016 for studies reporting the relationship between the presence of desmosomal protein gene mutations and the clinical features of ARVC. Two types of search terms were combined using the Boolean operator “and”: (1) “arrhythmogenic right ventricular dysplasia” OR “arrhythmogenic right ventricular cardiomyopathy” OR “ventricular dysplasia, right, arrhythmogenic” OR “right ventricular dysplasia, arrhythmogenic” OR “ARVC” OR “ARVD”; and (2) “plakophilin-2” OR “desmoplakin” OR “desmoglein-2” OR “desmocollin-2” OR “plakoglobin” OR “desmosome” OR “desmosomal”. We did not apply any language restrictions. In addition, we reviewed the reference lists, relevant journals, and conference abstracts for further searches.

Three independent authors (X-ZY, Z-WG and W-C) conducted the study selection and data extraction. The studies were first screened by reading the titles or abstracts to exclude the reviews, case reports, or irrelevant articles, and studies were then selected by reading the full text. Studies were considered eligible if they were: (1) cohorts of unrelated and consecutive ARVC patients where at least 2 desmosomal genes were sequenced, data on relatives were excluded from the pooled analysis; and (2) studies comparing the clinical features in ARVC patients with and without desmosomal gene mutations. If there were multiple publications, we included the study with the longest follow-up or the largest populations.

### Data extraction

For each study, the following data were recorded: the first author, publication year, country or region, study population, number of cases, number of genes sequenced, positive rate of desmosomal gene mutations, and incidence rate of each desmosomal gene. We also extracted the predefined clinical features that were most frequently reported in the final selected studies: onset age of ARVC, gender, and phenotypic features on the basis of international task force criteria (TFC): global or regional dysfunction and structural alterations, T wave inversion in right precordial leads (V_1–3_), epsilon wave in right precordial leads (V_1–3_), ventricular tachycardia of left bundle-branch morphology, and family history of ARVC. Any disagreements were resolved through discussion or by a fourth researcher (H–K).

### Statistical analyses

All of the statistical analyses were performed using Review Manager version 5.30 software (*the Nordic Cochrane Center, Rigshospitalet, Denmark*). Consistency was evaluated using the Cochrane Q test complemented with the *I*^*2*^ statistic, where 25% or less, 50%, and 75% or more indicated low, moderate, and high heterogeneity, respectively. The continuous variables were summarized as the mean ± standard deviation (SD), while the categorical variables were summarized as risk differences (RDs) and 95% confidence intervals (CIs). The data were pooled by the random-effects model, which was more conservative to evaluate risk estimates than the fixed-effects model. We pooled the predefined clinical features that were most frequently reported in the final selected studies (onset age of ARVC, gender, and phenotypic features on the basis of TFC). A meta-analysis of other clinical features including interventions and prognosis of ARVC (e.g., implantable cardioverter-defibrillator implantation (ICD), cardiac transplant, heart failure, and arrhythmic outcomes) was not possible because the features were inconsistently reported or not rigorously defined in the majority of the selected studies. A P value < 0.05 was considered statistically significant.

## Results

### Study selection

**Figure 1:**
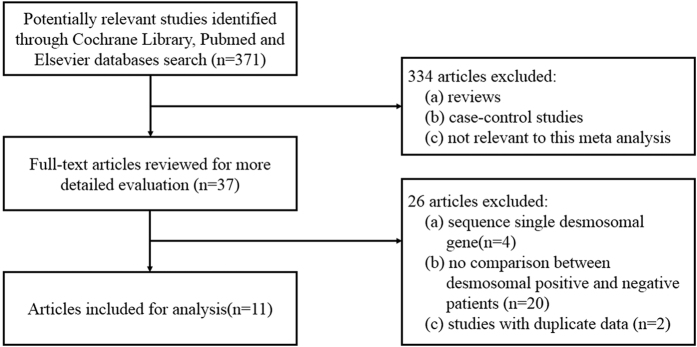
Flow chart of the study selection process.

As shown in Fig. 1, we initially retrieved a total of 371 studies through the electronic retrieval and the manual search after removing duplicates. After the first screening based on abstracts or titles, 334 studies were excluded because they were reviews, case-control studies, or studies that were not relevant to our analysis. The full texts of the 37 remaining studies were reviewed, and 26 of the 37 studies were excluded because they were (a) studies that sequenced only one of the desmosomal genes (n = 4); (b) studies that did not compare the clinical features in ARVC patients with and without desmosomal mutations (n = 20); and (c) studies that had duplicate data (n = 3), with 2 of the 3 excluded due to a smaller sample size^13^,^14^.

Finally, 11 studies comprising 1,113 participants were included in this meta-analysis^15^,^16^,^17^,^18^,^19^,^20^,^21^,^22^,^23^,^24^,^25^. The detailed characteristics of these studies are presented in Table 1. The diagnosis of patients with ARVC was made based on the original 1994 TFC^26^ and the revised 2010 TFC^27^. Four studies used the 1994 TFC^16^,^19^,^20^,^23^ and the 7 remaining studies were diagnosed according to the 2010 TFC^15^,^17^,^18^,^21^,^22^,^24^,^25^. In the pooled analysis, the percentage of individuals who were desmosomal-positive was 49.3% (95% CI: 39.6–59.0%).

### Demographic characteristics

#### Onset age of ARVC

**Figure 2:**
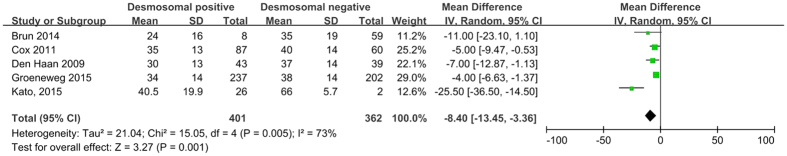
Forest plot of the relationship between the presence of desmosomal protein gene mutations and the onset age of ARVC. Abbreviations: ARVC = arrhythmogenic right ventricular cardiomyopathy; SD = standard deviation.

Several studies have reported a younger onset age in ARVC patients with desmosomal gene mutations^19^,^25^. In contrast, in the study by Cox *et al*.^18^, the presence of desmosomal gene mutations was not associated with an earlier onset age of ARVC. In addition, carriers with nonsense PKP2 mutations had onset at a later age than those with missense mutations^15^. In the pooled analysis, the average onset age for desmosomal-positive individuals was 32.7 ± 15.2 years, and the average onset age was 43.2 ± 13.3 years for desmosomal-negative individuals. There was a significant difference between these 2 groups (*P* = 0.001; Fig. 2), indicating that the presence of desmosomal gene mutations was associated with a younger onset age of ARVC.

#### Gender

**Figure 3:**
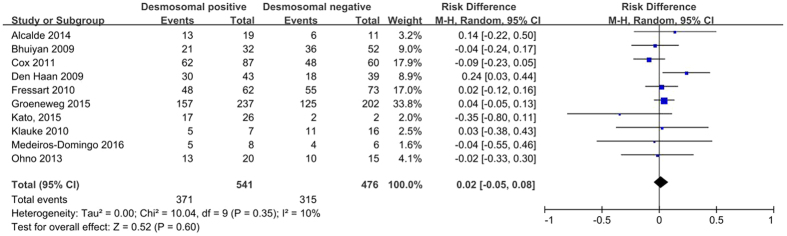
Forest plot of the proportion of male desmosomal-positive patients with ARVC compared with desmosomal-negative patients. Abbreviations: ARVC = arrhythmogenic right ventricular cardiomyopathy; CI = confidence interval; M-H = Mantel-Haenszel.

A higher prevalence of male patients is reported in the majority of ARVC cohorts^11^, and the male gender may be an independent predictor of appropriate ICD therapy^28^. In a study by Kato *et al*.^22^, the absence of desmosomal gene mutations was associated with a higher proportion of males, but other studies obtained contradictory results^15^,^16^,^18^,^19^,^21^,^23^,^24^,^25^. Overall, the pooled analysis showed no significant difference in the proportion of males between desmosomal-positive and desmosomal-negative patients (68.3% *versus* 68.9%; *P* = 0.60; Fig. 3).

### Phenotypic features on the basis of TFC

#### Structural and functional alterations

**Figure 4:**
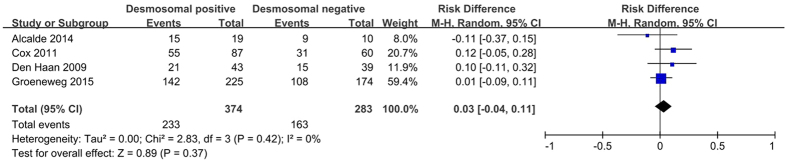
Forest plot of the proportion of structural and functional changes in desmosomal-positive patients compared with desmosomal-negative patients with ARVC. Abbreviations: ARVC = arrhythmogenic right ventricular cardiomyopathy; CI = confidence interval; M-H = Mantel-Haenszel.

Global or regional structural and functional alterations are mainly assessed by imaging examinations with various indexes, especially in the revised 2010 TFC^27^,^29^. Despite its low prevalence, the evaluation of ARVC accounts for a disproportionately high percentage of referrals for imaging examinations^29^. The structural and functional alterations presented in four included studies^15^,^18^,^19^,^21^ were evaluated by both echocardiography and cardiac magnetic resonance (CMR). Moreover, only one study performed a right ventricular angiography during phenotypic evaluation^21^. Given that each index is inconsistently reported across studies, we regarded all the major indexes together as a category. As shown in Fig. 4, there was no difference in the proportion of structural and functional alterations in patients with desmosomal gene mutations compared to those without mutations (63.5% *versus* 60.5%; *P* = 0.37).

#### Epsilon waves in right precordial leads

**Figure 5:**
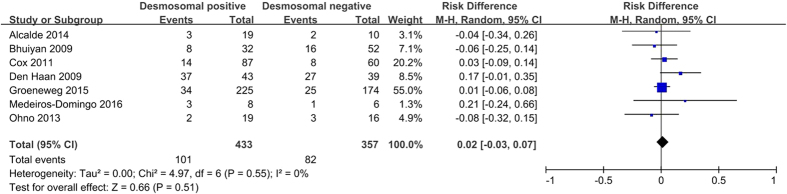
Forest plot of the proportion of subjects positive for epsilon waves in desmosomal-positive compared with desmosomal-negative patients with ARVC. Abbreviations: ARVC = arrhythmogenic right ventricular cardiomyopathy; CI = confidence interval; M-H = Mantel-Haenszel.

The epsilon waves is one of the characteristic clinical features of ARVC. It represents a delay in depolarization of the right ventricular (RV) free wall and outflow tract in patients with ARVC^30^. The pooled analysis showed no difference in the presence of epsilon waves between desmosomal-positive and desmosomal-negative individuals (29.4% *versus* 26.2%; *P* = 0.51; Fig. 5).

#### T wave inversion in right precordial leads

**Figure 6:**
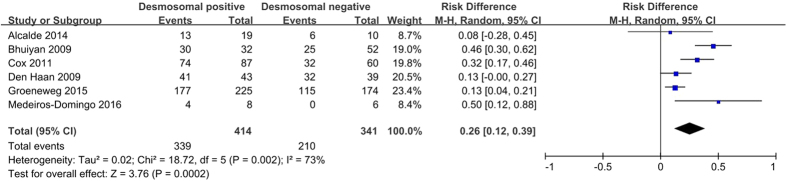
Forest plot of the proportion of negative T waves V_1–3_ in desmosomal-positive and desmosomal-negative patients with ARVC. Abbreviations: ARVC = arrhythmogenic right ventricular cardiomyopathy; CI = confidence interval; M-H = Mantel-Haenszel.

Electrocardiograms (ECGs) and signal-averaged ECGs are analyzed for depolarization and repolarization abnormalities, including T-wave inversions as the most common ECG alteration^10^. T wave inversion in V_1–3_ leads is demonstrated as one of the most sensitive and specific markers for the identification of desmosomal mutation carriers^16^,^20^. As shown in Fig. 6, the pooled analysis showed a higher incidence of T wave inversion in V_1–3_ leads in desmosomal-positive patients than in desmosomal-negative patients (78.5% *versus* 51.6%; *P* = 0.0002).

#### Ventricular tachycardia of left bundle-branch morphology

**Figure 7:**
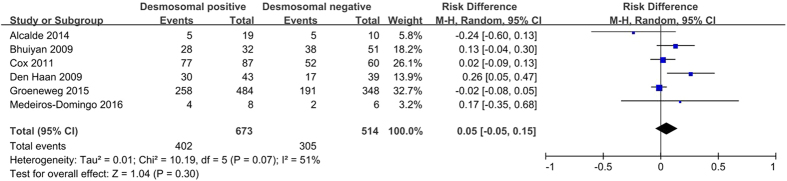
Forest plot of the proportion of ventricular arrhythmias of left bundle-branch morphology in desmosomal-positive compared with desmosomal-negative patients with ARVC. Abbreviations: ARVC = arrhythmogenic right ventricular cardiomyopathy; CI = confidence interval; M-H = Mantel-Haenszel.

Pathologically progressive fibrofatty replacement is thought to be responsible for ventricular arrhythmias. Electrophysiological studies are not included in the diagnostic criteria, but may be important for differential diagnosis, including RV outflow tract tachycardia^10^. Ventricular tachycardia of left bundle-branch morphology is the major index of ventricular arrhythmia in TFC. The meta-analysis showed no statistically significant difference in the presence of ventricular tachycardia of left bundle-branch morphology between desmosomal-positive and desmosomal-negative individuals (62.6% *versus* 57.2%; *P* = 0.30; Fig. 7).

#### Family history of ARVC

**Figure 8:**
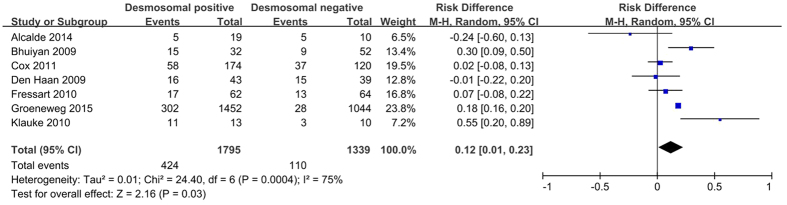
Forest plot of the proportion of a family history of ARVC in desmosomal-positive compared with desmosomal-negative patients with ARVC. Abbreviations: ARVC = arrhythmogenic right ventricular cardiomyopathy; CI = confidence interval; M-H = Mantel-Haenszel.

According to the study by Te Riele *et al*.^31^, one-third of first-degree relatives of ARVC probands could develop ARVC, especially siblings. Therefore, the fulfillment of TFC independent of family history is superior to conventional TFC for arrhythmic risk stratification of relatives^31^. Several studies have reported on the association of family history with the presence of desmosomal gene mutations, but the results remain contradictory. The pooled analysis showed a higher incidence of family history of ARVC in desmosomal-positive patients than in desmosomal-negative patients (39.5% *versus* 27.1%; *P* = 0.03; Fig. 8).

### Interventions and prognosis of ARVC

A meta-analysis of the interventions and prognosis of ARVC was not possible due to the limited phenotype data and high genetic heterogeneity. Therefore, we performed a descriptive analysis of the selected clinical features. Data on the frequency of ICD implantation in mutation carriers were contradictory. An increased prevalence of implanted ICDs was reported in the desmosomal-positive patients with ARVC^32^, but not in the desmosomal mutation carriers. In addition, several studies have reported the specific associations between individual mutations in the desmosomal genes and outcomes in patients with ARVC. In the study by van Tintelen *et al*.^33^, the presence of a PKP2 mutation was not associated with higher risks of endpoint events (documented sustained ventricular tachycardia episodes, ventricular fibrillation, appropriate ICD therapy, successful resuscitation and SCD). DSP mutation carriers were considerably more likely to develop heart failure, signs of left ventricular involvement and SCD^14^,^34^, but these results are contradictory for DSG2 mutation carriers^14^,^20^.

## Discussion

Studies on the genotype-phenotype relationship in ARVC have improved the understanding of the molecular mechanisms leading to ARVC and the value of genetic counseling for ARVC. However, the genetic substrate, pathogenesis and clinical diagnosis of ARVC patients (especially for asymptomatic gene mutation carriers) are still poorly understood. Previous studies on the genotype-phenotype relationship in patients with ARVC are largely limited because of the small population size as well as the genetic and clinical heterogeneity. Therefore, the clinical significance of the genotype is obscure and the value of genetic analysis in developing a clinical strategy is still controversial. Our meta-analysis first expanded the sample size to explore the relationship between the presence of desmosomal gene mutations and selected clinical features.

The impact of sex on clinical course in ARVC patients is still unclear. Our analysis showed that there was no difference in the proportion of males between desmosomal-positive and desmosomal-negative patients, which is consistent with the majority of studies^20^,^21^,^23^,^25^. In addition, the incidence of desmosomal gene mutations was associated with a younger onset age of ARVC, and the initial symptoms in some young probands were more severe^19^,^25^. The presence of a PKP2 mutation is also associated with an earlier onset of symptoms and arrhythmias^9^. In addition, many previous studies have indicated that the presence of both single and multiple desmosomal gene mutations is associated with a younger onset age of ARVC symptoms and ventricular tachycardia^14^,^19^,^33^,^35^. Multiple desmosomal gene mutations (compound heterozygosity, digenic or trigenic heterozygosity or homozygosity) were identified in 16% of ARVC-causing desmosomal gene mutation carriers and have a close association with a younger onset age and arrhythmia^35^. Therefore, compound or digenic desmosomal mutations are listed as independent risk factors for ARVC according to the 2015 European Society of Cardiology (ESC) Guidelines for the management of patients with ventricular arrhythmia and the prevention of SCD^36^.

For the phenotypic features based on TFC, the presence of desmosomal gene mutations was associated with a higher proportion of T wave inversion in V_1–3_ leads (which occurred more often in PKP2 mutation carriers)^33^ and a family history of ARVC. ARVC is characterized by a propensity towards arrhythmia exceeding the degree of ventricular dysfunction, and T wave inversion in the V_1–3_ leads is a major index of repolarization according to the revised 2010 TFC^27^. Desmosomal proteins are involved in cell-cell adhesion and help maintain myocyte integrity^3^. It has been reported that the expression of connexin 43 is significantly decreased by desmosomal genes (PKP2 and JUP) mutations and is associated with the development of arrhythmia in ARVC patients^37^,^38^. The extent of negative T-waves can help estimate the amount of RV electroanatomic scar (EAS) and predict EAS-related arrhythmic risk^39^. Thus, desmosomal mutation may lead to abnormal repolarization of cardiomyocytes, resulting in T wave inversion of the right precordial lead on 12-leads ECG.

However, the relevant incidence of structural or functional alterations, epsilon waves, and ventricular tachycardia of left bundle-branch morphology had no associations with the presence of desmosomal gene mutations. This may because ARVC phenotypic expression is a prerequisite for the occurrence of life-threatening arrhythmias in desmosomal gene mutation carriers^40^ or the 3 aforementioned phenotypes may be more severe in those patients with non-desmosomal mutations such as transmembrane protein 43 and phospholamban mutations.

Due to the widely clinical application of next-generation sequencing, massive candidate gene screening can identify various mutations that may have not been identified by Sanger technology, and the genetic basis of ARVC is now better understood. Although guidelines and expert consensus suggested that comprehensive or targeted genetic screening can be useful for patients satisfying TFC^41^,^42^, genetic testing is presently handicapped by low yields, unclear clinical impact, and high background noise rates that make ARVC genetic test interpretations still challenging^43^. Thus, we must emphasize the necessity of a specialist in clinical practice to explain the implications of the genetic abnormality. Meanwhile, precise genotype-phenotype studies are also very important. Consistent differences between desmosomal-positive and desmosomal-negative patients suggest that, in spite of the inconsistency of each gene, desmosomal genes might be considered as a clinical entity. In accordance with our meta-analysis, desmosomal gene mutations were associated with a younger onset age, a higher prevalence of negative T waves V_1–3_ and a family history of ARVC. This information may help improve our understanding of the different clinical manifestations of ARVC and differential diagnosis, prognosis and treatment of patients with ARVC.

Our study also had several potential limitations. First, there were substantial differences regarding study design, case ascertainment and the assessment of clinical manifestations. The heterogeneous nature of ARVC and the inconsistency of study designs precluded the establishment of more precise genotype–phenotype relationships. For example, in this meta-analysis, the structural and functional alterations presented in four included studies are evaluated by both echocardiography and CMR, without further comparison in each index. This may cause bias and confusion, thus further studies should compare indicators of structural and functional alterations based on TFC in detail. Second, several factors might influence disease expression. For example, some mutations were unique to each family, ethnic background, and environmental factor and had low penetrance. Nevertheless, these results were stable in the sensitivity analysis. Third, because of the limited data, the diagnosis criteria of ARVC was inconsistent across studies, thus more studies with consistent diagnosis criteria will be needed in the future. Finally, a meta-analysis of specific associations between individual desmosomal gene mutations and clinical phenotypes was not possible due to limited data. Large cohort studies with a reasonable design and grouping that examine the significance among genotype, risk stratification and prognosis are also needed. The underlying mechanisms of the desmosomal genes that affects the clinical features of ARVC also deserve further investigation.

## Conclusions

In summary, our meta-analysis first demonstrated that the presence of desmosomal gene mutations was associated with several clinical features, including a younger onset age of ARVC, a higher prevalence of negative T waves V_1–3_ and a family history of ARVC. The clinical application of genetic testing and the interpretation of genetic variations for ARVC is still challenging. Owing to the heterogeneity of ARVC, large-scale studies on the genotype-phenotype relationship with more detailed indicators in patients with ARVC are required to define the value of genetic screening for clinical practice.

## Additional Information

**How to cite this article:** Xu, Z. *et al*. Genotype-phenotype relationship in patients with arrhythmogenic right ventricular cardiomyopathy caused by desmosomal gene mutations: A systematic review and meta-analysis. *Sci. Rep.* **7**, 41387; doi: 10.1038/srep41387 (2017).

**Publisher's note:** Springer Nature remains neutral with regard to jurisdictional claims in published maps and institutional affiliations.

## Figures and Tables

**Table 1:** Characteristics of the 11 studies included in the pooled analysis.

First Author, Year	Region	Patients (n)	Genes (n)	Desmosomal positive (%)	PKP2 (%)	JUP (%)	DSG2 (%)	DSC2 (%)	DSP (%)
Bhuiyan, 2009	Netherlands	84	3	32 (38.1)	29.8	—	4.8	1.2	—
Den Haan, 2009	United States	82	5	43 (52.4)	—	—	—	—	—
Fressart, 2010	France	135	5	62 (45.9)	29.6	0	10.4	1.5	4.4
Klauke, 2010	Germany	23	5	13 (56.5)	26.1	0	8.7	8.7	8.7
Cox, 2011	Netherlands	147	5	89 (60.5)	53.1	0	3.4	1.4	0.7
Ohno, 2013	Japan	35	4	20 (57.1)	25.7	—	5.7	2.9	14.3
Alcalde, 2014	Spain	30	6	19 (63.3)	43.3	0	10.0	3.3	6.7
Brun, 2014	United States; Italy	67	5	8 (11.9)	7.5	0	3.0	0	1.5
Groeneweg, 2015	United States; Netherlands	439	7	237 (54.0)	46.0	0.4	3.9	1.1	2.5
Kato, 2015	Japan	57	8	26 (45.6)	19.3	—	12.3	1.8	5.3
Medeiros-Domingo, 2016	Switzerland	14	96	8 (57.1)	7.1	7.1	35.7	7.1	7.1

**Abbreviations:** JUP = plakoglobin; PKP2 = plakophilin-2; DSP = desmoplakin; DSG2 = desmoglein-2; DSC2 = desmocollin-2.
